# Adsorption of Potentially Toxic Metals from Mono and Multi-Metal Systems Using Groundnut and Shea Nut Shell Biochars

**DOI:** 10.5696/2156-9614-8.18.16

**Published:** 2018-06-11

**Authors:** Abudu Ballu Duwiejuah, Samuel Jerry Cobbina, Albert Kojo Quainoo, Abdul Halim Abubakari, Noel Bakobie

**Affiliations:** 1 Department of Biotechnology, Faculty of Agriculture, University for Development Studies, Tamale, Ghana; 2 Department of Ecotourism and Environmental Management, Faculty of Natural Resources and Environment, University for Development Studies, Tamale, Ghana; 3 Department of Horticulture, Faculty of Agriculture, University for Development Studies, Tamale, Ghana

**Keywords:** adsorption isotherms, biochars, removal efficiency, simultaneous adsorption, toxic metals

## Abstract

**Background.:**

Adsorption is a unique and promising method for the removal of trace metals from an aqueous environment using cost-effective and readily available biochars.

**Objective.:**

The present study examined mono and simultaneous adsorption of cadmium (Cd), mercury (Hg) and lead (Pb) onto biochars produced at pyrolysis temperatures of 350 ± 5°C and 700 ± 5°C.

**Methods.:**

Fifty mg/l of trace metal ions with 2 g/50 ml of adsorbent dosage were leached at constant room temperature of 24 ± 0.5°C in the laboratory with a constant contact time of 72 minutes. A total of 126 elutes were obtained from the batch experiments and conveyed to the Ecological Laboratory at University of Ghana for the analysis.

**Results.:**

In the mono-component system of Cd, Hg and Pb, removal efficiency was almost 100% using groundnut, shea nut shell, and a combination of groundnut and shea nut shell biochars. The experiment showed that shea nut shell biochars have the strongest affinity for trace metal ions in the mono aqueous phase. In the binary system, the removal efficiency was over 99.60% for cadmium and 100% for mercury. The ternary experiment showed an order of adsorption of Pb^2+^ > Hg^2+^ > Cd^2+^ for Cd, Hg and Pb ions onto groundnut and shea nut shells biochars. Fast pyrolysis temperatures and some types of biochar showed a slight increase in the adsorption efficiency of metal ions, but the increase was not statistically significant (p > 0.05).

**Conclusions.:**

The study revealed that the Langmuir adsorption isotherm was the best fit model for trace metal ion adsorption onto biochars in the batch experiment. The interactive effects of binary and ternary metal systems onto biochars are antagonistic and synergistic in nature. Based on these results, it is recommended that further competitive adsorption studies of these biochars should be undertaken for accurate estimation of adsorption in natural environments.

**Competing Interests.:**

The authors declare no competing financial interests.

## Introduction

Biochars are carbonaceous substances from the pyrolysis of biomass in a limited oxygen condition that can be utilized for long-term carbon sequestration and are safe for the environment.^1^,^2^ The utilization of biochar has attracted attention in agricultural waste recycling, global warming mitigation, pollution remediation and soil fertility improvement.^3^

Heavy metals have become a global environmental and public health concern due to their toxicity, bioaccumulation in the human body and food chain, carcinogenicity, and mutagenesis in living organisms.^4^ Exposure to cadmium (Cd) can cause acute and chronic conditions of the kidneys, cardiovascular system, liver and nervous system.^5^ Exposure to mercury (Hg) has been linked to blindness, cerebral palsy, deafness and mental retardation, especially in children exposed in utero.^6^ Lead (Pb) toxicity can cause decreases in hemoglobin production, joint, kidney, reproductive and cardiovascular system conditions and long-term damage to the central and peripheral nervous systems.^7^

Biochars have high adsorption capacities for trace metals and organic compounds from aqueous solutions when compared with activated carbon.^8^ Generally, activated carbon is more expensive than biochar. The estimated cost of biochar is US$246 per ton, about one-sixth the cost of commercially available activated carbon, which costs approximately US$1500 per ton.^9^ In addition to cost-effectiveness, biochar requires less energy than activated carbon and can be easily used without further modification or activation. The use of activated carbon as an adsorbent can be out of reach of poor and developing countries due to its high cost.

Consequently, there is an urgent need to develop a novel method that will not only be cost-effective, but practically workable. Adsorption on to a low-cost adsorbent material is a better choice to overcome the limitations of other methods. The removal of trace metals from an aqueous environment by means of adsorption is an efficient method due to its effectiveness at low concentrations.^10^,^11^

Biochar can be obtained from the direct burning of precursors due to the wide availability of its feed stocks. A wide range of waste biomass has been used to produce biochar and used as an adsorbent for the removal of pollutants from waste water.^12^ Cheap sources of biomass such as agricultural waste used in the production of biochar cost around US$4 per gigajoule to produce, with the majority of this cost associated with heating and machinery.^13^ The use of agricultural wastes as adsorbents for the removal of trace metals has some advantages that include availability in large quantities, eco-friendliness, cost-effectiveness and renewability.^14^

Many studies have sought to find economically efficient and unconventional adsorbents produced from agricultural/plant wastes.^15^ For instance, ground shea nut shells have proven to be effective in the removal of trace metals, particularly magnesium (Mn), iron (Fe), zinc (Zn) and copper (Cu) in soils using lettuce as a test crop.^16^ There has been a gradual increase in the utilization of agricultural waste as biochar feed stock as part of a waste management strategy, mainly due to its relatively low cost and greater availability. Groundnut and shea nut shells were used as feed stocks in the present study due to their availability, cost-effectiveness, and effectiveness as a waste utilization method, aided by the presence of lignocellulosic biomass (cellulose, hemicellulose and lignin) which is a characteristic for successful heavy-metal adsorption. Agricultural wastes contain acidic groups such as carboxylic and phenolic groups, which are characteristic of successful heavy metal adsorption products.^17^ Hence, there is a need for cost-effective biochars for adsorption of trace metals in the aqueous phase.

## Methods

### Chemicals and materials

Cadmium chloride (CdCl_2_, grade; anhydrous), mercury chloride (HgCl_2,_ grade; American Chemical Society reagent) and lead nitrate (Pb (NO_3_)_2_ grade) were obtained from the Spanish Laboratory Complex at the University for Development Studies. Stock solutions of 1000 mg/l each of Cd, Pb and Hg were prepared by dissolving 1.68 g of cadmium chloride, 1.35 g of mercury chloride, and 1.60 g of lead nitrate in deionized water. The groundnut and shea nut shells were collected from Phebsigu and Shishegu in the Tamale Metropolis, Ghana. The groundnut and shea nut shells (feed stocks) were charred at 350 ± 5°C for 60 minute and 180 minute, respectively, and 700 ± 5°C for 45 minute and 90 minute for groundnut and shea nut shells, respectively. The experimental biochars were produced and are abbreviated as follows:

- GB350 and GB700: groundnut shell biochar produced at 350 ± 5°C and 700 ± 5°C, respectively
- SB350 and GB700: shea nut shell biochar produced at 350 ± 5°C and 700 ± 5°C, respectively
- MB350 and MB700: groundnut and shea nut shell biochar produced at 350 ± 5°C and 700 ± 5°C, respectively

**Abbreviations**
*B*: Constant related to heat of sorption
*R_i_*: Relative adsorption capacity

Biochar production took place at the Spanish Laboratory Complex at the University for Development Studies, Nyankpala Campus under limited oxygen conditions in a muffle furnace. The biochars were cooled at room temperature, crushed and sieved through a 2 mm standard mesh sieve.

### Adsorption experiment and trace metals analysis

Serial dilutions of the stock solutions for Cd, Hg and Pb (one-, two-, and five-fold dilutions) were carried out to obtain leaching solutions in accordance with the procedure described by Saveyn et al.^18^ The thresholds of the toxic metals form the maximum contamination limits: 0.04 mg/l for Cd, 0.10 mg/l for Hg, and 0.10 mg/l for Pb (one-fold), 0.08 mg/l for Cd, 0.20 mg/l for Hg, and 0.50 mg/l for Pb (two-fold), and 0.20 mg/l for Cd, 0.50 mg/l for Hg, and 0.50 mg/l for Pb (five-fold) from the aqueous phase. These different maximum contamination limits were combined to form the binary and ternary phase as their source contamination varied.

The aqueous solution pH values were in the acidic range of 3.25 to 4.63 pH, 2 g biochar with particles size ≤ 2 mm, laboratory temperature of 24 ± 0.5°C and constant contact time of 72 minutes under a constant flow rate. The adsorption experiments were performed using separatory funnels to leach 50 mg/l of trace metals regardless of the variant concentrations. Test solutions were allowed to gravitationally leach from the top of experiment funnels. Elutes were then automatically sampled immediately after the whole retention test and then filtered using Whatman's qualitative filter paper with a particle retention size of 125 mm Ø. Plastic bottles with volume capacity of 35 ml and a screw cap were used to collect the elute and conveyed to the laboratory. Elutes were kept in an ice chest and transported to the Ecological Laboratory at the University of Ghana for the analysis. The analysis of trace metals was performed using atomic absorption spectrophotometry (PinAAcle 900T). The detection limits of the metal ions were 0.0008 mg/l for Cd, 0.0001 mg/l for Hg and 0.003 mg/l for Pb.

### Adsorption models

The adsorption efficiency of trace metals (Cd, Hg and Pb), Qe (mg/g) was calculated using Equation 1:

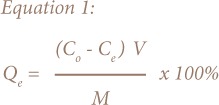
Where C_o_ and C_e_ are the initial and final concentrations (mg/l), respectively, M is the adsorbent dosage (g), and V is the volume of solution (l).

This study employed Langmuir (Equations 2 and 3) and Freundlich (Equation 4) models for fitting the equilibrium data.^19^,^20^

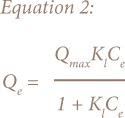


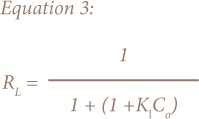


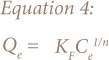



Where Q_max_ is the maximum adsorption capacity, K_l_ represents the Langmuir constant (l mg^−1^), K_F_ (l g^−1^) and n (dimensionless).

Where R_L_ is a dimensionless constant denoted as equilibrium parameter or separation factor C_0_ = initial concentration of absorbate, K_L_ = the constant that is related to the adsorption energy (Langmuir constant). R_L_ value designates the nature of adsorption to be either, linear if R_L_ = 1, favourable if 0 < R_L_< 1, unfavourable if R_L_ > 1 and irreversible if R_L_ = 0.

The derivation of the Temkin adsorption isotherm is characterized by a uniform distribution of binding energies by plotting quantity sorbed Q_e_ against lnC_e_ and constants were calculated from the slope and intercept. The model is given by the following equations:^21^

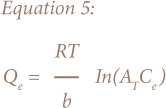


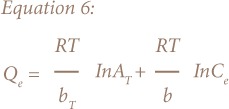


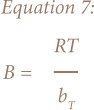


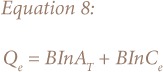



Where A_T_ = Temkin isotherm equilibrium binding constant (L/g); b_T_ = Temkin adsorption isotherm constant; R = universal gas constant (8.314 J/mol/K); T = temperature at 297 K; and B = constant related to heat of sorption (J/mol).

### Interactive behavior of the trace metals

The selectivity of adsorbents for Cd, Hg and Pb in both binary and tertiary mixtures was determined in terms of relative metal i adsorption, (R_i_) (%), as defined in Chang and Chen.^22^ The percentage R_i_ determines whether the combinations in binary and tertiary systems are antagonistic or synergistic in nature.


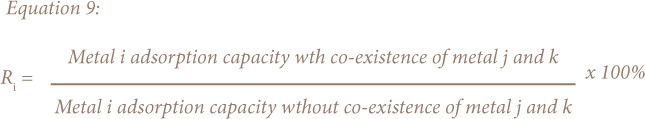


If R_i_ > 100%, this indicates that the interactive effect of a mixture of metals (say, *j* and *k*) is synergistic, If R_i_ < 100%, this indicates antagonistic behavior and R_i_ = 100% indicates non-interactive behavior.

### Statistical analysis

The effects of pyrolysis temperature, type of biochar, aqueous phase pH and different maximum contamination limits on the adsorption capacity of Cd, Hg and Pb ions onto biochar were statistically analyzed using the Statistical Package for Social Science software (SPSS version 16). Differences were considered be significant at *p* < 0.05.

## Results

### Adsorption of mono-component system onto biochars

The removal efficiencies of mono metals by biochars are presented in Table 1. Cadmium removal efficiency in a mono-component system by biochars was greater than 99.50%. Mercury removal efficiency by biochars in a mono-component system with different maximum contamination limits was almost 100% *(Table 1)*. The removal efficiency of Pb in a mono-component system by biochars was greater than 99.10% *(Table 1)*. At 350 ± 5°C, the adsorption was less than 100% for Pb at the different maximum contamination limits of the mono aqueous phase compared to the binary and ternary aqueous phases *(Table 1)*.

**Table 1:** Adsorption Efficiency of Mono Metals by Biochars

**Pyrolysis**	**Metal**	**Initial concentration (mg/l)**	**Groundnut shell (%)**	**Shea nut shell (%)**	**Groundnut and shea nut shells (%)**
350 ± 5°C	Cd	0.04	100	100	100
		0.08	99.99	100	99.59
		0.20	100	99.98	99.92
700 ± 5°C	Cd	0.04	100	100	100
		0.08	99.67	100	100
		0.20	99.93	100	100
350 ± 5°C	Hg	0.10	99.99	100	100
		0.20	100	100	100
		0.50	100	100	100
700 ± 5°C	Hg	0.10	100	100	100
		0.20	100	100	100
		0.50	100	100	100
350 ± 5°C	Pb	0.10	99.12	100	99.47
		0.20	100	100	99.68
		0.50	100	100	99.84
700 ± 5°C	Pb	0.10	100	100	100
		0.20	100	100	100
		0.50	100	100	100

### Adsorption of binary-component system onto biochars

In the binary system of Cd and Hg, Cd removal efficiency was greater than 99.60% and Hg removal efficiency was 100% *(Table 2).* The removal efficiencies of Cd and Pb in a binary system by biochars are presented in Table 2. In the binary system of Hg and Pb, Hg removal efficiency was almost 100% at the various maximum concentration limits *(Table 2).*

**Table 2:** Removal Efficiency of Binary Metals by Biochars

**Pyrolysis**	**Type of biochar**	**Groundnut shell**		**Shea nut shell**		**Groundnut and shea nut shells**	
	**Cd : Hg Initial concentration (mg/l)**	**Cd (%)**	**Hg (%)**	**Cd (%)**	**Hg (%)**	**Cd (%)**	**Hg (%)**
***350 ± 5°C***	0.04 : 0.10	99.67	100	100	100	100	100
	0.08 : 0.20	99.98	100	100	100	100	100
	0.20 : 0.50	99.96	100	100	100	100	100
***700 ± 5°C***	0.04 : 0.10	100	100	100	100	100	100
	0.08 : 0.20	99.99	100	100	100	99.95	100
	0.20 : 0.50	99.98	100	100	100	100	100
**Pyrolysis**	**Cd: Pb Initial concentration (mg/l)**	**Cd (%)**	**Pb (%)**	**Cd (%)**	**Pb (%)**	**Cd (%)**	**Pb (%)**
***350 ± 5°C***	0.04 : 0.10	99.70	100	100	100	100	100
	0.08 : 0.20	99.70	100	100	100	99.61	100
	0.20 : 0.50	100	100	100	100	99.90	100
***700 ± 5°C***	0.04 : 0.10	100	100	100	99.04	100	98.20
	0.08 : 0.20	100	100	100	100	100	100
	0.20 : 0.50	100	100	100	100	100	100
**Pyrolysis**	**Hg : Pb Initial concentration (mg/l)**	**Hg (%)**	**Pb (%)**	**Hg (%)**	**Pb (%)**	**Hg (%)**	**Pb (%)**
***350 ± 5°C***	0.10 : 0.10	99.99	100	100	99.14	100	98.84
	0.20 : 0.20	99.92	100	100	100	100	100
	0.50 : 0.50	99.84	100	100	100	100	100
***700 ± 5°C***	0.10 : 0.10	100	100	100	98.72	99.99	100
	0.20 : 0.20	100	100	100	100	100	100
	0.50 : 0.50	100	100	100	100	100	100

### Adsorption of ternary-component system onto biochars

The removal efficiency of Cd, Hg and Pb ions by groundnut and shea nut shells biochars produced at 350 ± 5°C and 700 ± 5°C was greater than 97.50% for the prepared aqueous phase with concentrations of 0.04 : 0.10 : 0.10 mg/l, 0.08 : 0:20 : 0.20 mg/l and 0.20 : 0.50 : 0.50 mg/l, respectively *(Table 3)*

**Table 3:** Removal Efficiency of Ternary Metals by Biochars

		**Initial concentrations (mg/l)**		
**350 ± 5°C** **Biochar**	**Ions**	**Cd: Hg: Pb** **0.04: 0.10 : 0.10**	**Cd: Hg: Pb** **0.08 : 0.20 : 0.20**	**Cd: Hg: Pb** **0.20 : 0.50 : 0.50**
***Groundnut shell***	Cd	99.05^*^	99.46	99.69
	Hg	99.98	100	100
	Pb	100	100	99.90
***Shea nut shell***	Cd	100	100	100
	Hg	100	99.98	99.95
	Pb	97.50	100	100
***Groundnut and shea nut shells***	Cd	100	99.95	99.90
	Hg	100	99.98	99.96
	Pb	98.80	100	100
**700±5°C** **Biochar**	**Ions**	**Cd: Hg: Pb** **0.04 : 0.10 : 0.10**	**Cd: Hg: Pb** **0.08 : 0:20 : 0.20**	**Cd: Hg: Pb** **0.08 : 0:20 : 050**
***Groundnut shell***	Cd	100	99.99	99.98
	Hg	99.96	100	100
	Pb	100	100	100
***Shea nut shell***	Cd	100	99.99	100
	Hg	98.18	100	100
	Pb	98.46	100	100
***Groundnut and shea nut shells***	Cd	100	99.94	99.85
	Hg	99.80	100	100
	Pb	98.40	100	100

^*^all figures removal efficiency %

### Adsorption performance of biochars produced at 350 ± 5°C and 700 ± 5°C

The present study shows that shea nut shell-based biochar has a greater affinity for the studied trace metals in the mono, binary and ternary systems. The study revealed biochars produced at 700 ± 5°C have greater affinity for metals in the aqueous phase, particularly in binary and ternary systems compared to biochars produced at 350 ± 5°C (slow pyrolysis). Fast pyrolysis temperatures and some types of biochar showed a slight increase in the adsorption efficiency of metal ions, but this increase was not statistically significant (p > 0.05).

### Influence of aqueous phase pH on the adsorption capacity of biochars

Generally, pH values of the aqueous solution were in the acidic range of less than 5 pH units for the mono system, binary system and ternary system at one-, two- and five-fold concentration limits *(Table 4).* The pH decreased in the prepared aqueous phase at concentrations in the order of one-fold > two-fold > five-fold for mono, binary and ternary systems. Generally, there were no significant differences (*p* > 0.05) in terms of difference in the pH of the aqueous phase in adsorption of metal ions regardless of the component system or fold of the contamination limits.

**Table 4:** pH of Mono, Binary and Ternary Aqueous Phase

**Metal**	**pH for one-fold concentration limit**	**pH for two-fold concentration limit**	**pH for five-fold concentration limit**
***Cd***	4.63	3.91	3.58
***Hg***	3.42	3.39	3.36
***Pb***	3.85	3.38	3.36
***Cd : Hg***	3.31	3.40	3.41
***Cd : Pb***	3.63	3.25	3.31
***Hg : Pb***	3.39	3.33	3.39
***Cd : Hg : Pb***	3.39	3.28	3.35

### Langmuir, Freundlich and Temkin isotherms

The maximum sorption capacity corresponding to complete monolayer coverage showed that biochars produced at 350 ± 5°C had a mass capacity for Cd^2+^ (0.22 mg/g for MB350), Pb^2+^ (0.35 mg/g for SB350), Hg^2+^ (0.14 mg/g for GB350) and Hg^2+^ (0.87 mg/g for SB350), while biochars produced at 700 ± 5°C had a mass capacity for Cd^2+^ (0.18 mg/g for GB700) and Hg^2+^ (0.55 mg/g for SB700) in a mono-component system *(Table 5)*. The correlation coefficients (R^2^) derived from the Freundlich equations ranged from 0 to 1, indicating that few data fit this model well *(Table 5)*. Generally, most of the co-efficient correlation values, regardless of batch systems, were below 0.50 (*Table 6)*. The constant related to heat of sorption (B) values ranged from −0.00 to 1.02 KJ/mol for biochars produced at 350 ± 5°C, and at 700 ± 5°C, B values ranged from −0.01 to 1.02 KJ/mol for adsorbate in mono, binary and ternary systems *(Table 6)*. The Temkin isotherm equilibrium binding constant (A_T_) values ranged from 2.23 to 3.19 L/mg for biochars produced at 350 ± 5°C and between 2.51 to 3.67 L/mg at 700 ± 5°C for the absorbate in mono, binary and ternary systems *(Table 6).*

**Table 5:** Adsorption Isotherm Parameters Based on Langmuir and Freundlich Models

**Absorbent**	**Absorbate**	**Langmuir isotherm**			**Freundlich isotherm**		
		**Q_max_ (mg/g)**	**R_L_**	**R^2^**	**1/n**	**K_F_ (mg/g)**	**R^2^**
***MB350***	**Cd**	0.22	1 × 10^−4^	0.89	−0.11	0.04	0.53
***MB350***	**Pb**	0.36	2.2 × 10^−4^	0.56	−0.16	0.10	0.65
***GB350***	**Hg**	0.14	−3 × 10^−6^	0.43	0.03	0.31	0.00
***SB350***	**Hg**	0.87	1 × 10^−5^	0.75	0.63	515.82	0.82
***GB350***	**Cd** + Pb	0.09	3 × 10^−4^	0.95	0.16	0.21	0.93
***MB350***	**Cd** + Pb	0.08	0.00	1.00	0.01	0.09	0.01
***MB350***	**Hg** + Cd	0.89	1 × 10^−5^	0.42	0.32	8.83	0.11
***SB350***	**Hg** + Cd	0.56	3 × 10^−6^	0.91	−0.08	0.11	0.49
***GB350***	**Hg** + Cd	0.09	−4 × 10^−6^	0.93	−0.19	0.02	0.10
***GB350***	**Pb** + Hg	0.56	2.2 × 10^−4^	0.89	−0.12	0.11	0.51
***GB350***	**Hg** + Pb	0.48	−3 × 10^−6^	1.00	0.01	0.23	0.01
***SB350***	**Hg** + Pb	0.04	0.00	0.28	−0.08	0.10	0.68
***MB350***	**Hg** + Pb	0.5	3 × 10^−6^	0.57	−0.09	0.10	0.63
***MB350***	**Hg** + Cd + Pb	0.09	−1 × 10^−6^	0.99	−0.58	0.00	0.54
***SB350***	**Hg** + Cd + Pb	1.12	1.3 × 10^−5^	0.99	−0.09	0.11	0.49
***GB350***	**Cd** + Hg + Pb	0.05	−1.3 × 10^−4^	0.69	0.46	2.03	0.71
***MB350***	**Cd** + Hg + Pb	0.22	1.1 × 10^−4^	0.89	0.19	0.05	0.56
***GB350***	**Hg** + Cd + Pb	0.10	−1 × 10^−6^	1.00	0.09	0.44	0.62
***GB700***	**Cd**	0.18	9 × 10^−6^	0.89	−0.10	0.04	0.55
***SB700***	**Hg**	0.55	3.3 × 10^−6^	0.94	−0.08	0.11	0.55
***GB700***	**Cd** + Hg	0.22	3.3 × 10^−5^	0.90	−0.02	0.04	0.05
***MB700***	**Cd** + Hg	0.22	2.2 × 10^−4^	0.90	−0.11	0.04	0.53
***SB700***	**Hg** + Pb	−0.17	−2 × 10^−5^	0.99	2.28	1.92 × 10^11^	0.96
***MB700***	**Hg** + Pb	−0.08	−2 × 10^−5^	0.91	4.11	2.86 × 10	1.00
***GB700***	**Cd** + Hg + Pb	0.08	−4 × 10^−5^	0.87	−0.12	0.04	0.72
***MB700***	**Cd** + Hg + Pb	0.22	2.2 × 10^−4^	0.93	−0.11	0.05	0.51

Abbreviations: GB350, Groundnut shell biochar produced at 350±5°C; GB700, Groundnut shell biochar produced at 700±5°C; SB350, Shea nut shell biochar produced at 350±5°C; SB700, Shea nut shell biochar produced at 700±°C; MB350, Groundnut and shea nut shell biochar produced at 350±5°C; MB700, Groundnut and shea nut shell biochar produced at 700±5°C; R^2^, Correlation coefficient

**Table 6:** Temkin Isotherm Constants for Adsorption of Metal Ions

**Absorbent**	**Absorbate**	**Estimate parameters of R^2^ Temkin Isotherm**		
		**A_T_ (L/mg)**	**B**	**R^2^**
***MB350***	**Cd**	3.16	−0.01	0.34
***MB350***	**Pb**	2.46	−0.03	0.45
***GB350***	**Hg**	2.69	0.05	0.05
***SB350***	**Hg**	2.66	0.17	0.94
***GB350***	**Cd** : Pb	3.19	0.02	1.00
***MB350***	**Cd** : Pb	2.80	0.004	0.08
***MB350***	**Hg** : Cd	2.64	0.12	0.24
***SB350***	**Hg** : Cd	3.13	−0.02	0.36
***GB350***	**Hg** : Cd	3.00	−0.02	0.01
***GB350***	**Pb** : Hg	2.23	−0.02	0.31
***GB350***	**Hg** : Pb	3.04	−0.004	0.01
***SB350***	**Hg** : Pb	3.00	−0.02	0.48
***MB350***	**Hg** : Pb	2.86	−0.02	0.43
***MB350***	**Hg** : Cd : Pb	2.72	−0.12	0.35
***SB350***	**Hg** : Cd : Pb	3.19	−0.02	0.29
***GB350***	**Cd** : Hg : Pb	2.59	−0.07	0.91
***MB350***	**Cd** : Hg : Pb	3.13	−0.01	0.34
***GB350***	**Hg** : Cd : Pb	3.03	0.03	0.8
***GB700***	**Cd**	3.25	−0.01	0.34
***SB700***	**Hg**	3.16	−0.02	0.35
***GB700***	**Cd** : Hg	3.67	−0.01	0.36
***MB700***	**Cd** : Hg	3.06	−0.01	0.34
***SB700***	**Hg** : Pb	2.69	0.55	0.86
***MB700***	**Hg** : Pb	2.72	1.02	0.98
***GB700***	**Cd** : Hg : Pb	2.51	−0.01	0.53
***MB700***	**Cd** : Hg : Pb	3.13	−0.01	0.32

Abbreviations: GB350, Groundnut shell biochar produced at 350±5°C; GB700, Groundnut shell biochar produced at 700±5°C; SB350, Shea nut shell biochar produced at 350±5°C; SB700, Shea nut shell biochar produced at 700±°C; MB350, Groundnut and shea nut shell biochar produced at 350±5°C; MB700, Groundnut and shea nut shell biochar produced at 700±5°C

### Interactive behavior of cadmium, mercury and lead ions in binary and ternary systems

Some of the R_i_ values were less than 100% in binary and ternary metal systems and others were greater than 100%, especially for Hg ions in the mixtures that interacted with biochars produced at 350 ± 5°C and 700 ± 5°C *(Tables 7 and 8)*.

**Table 7:** Interactive Effect of Mixture of Metal Ions in the Binary and Ternary Systems onto Biochars Produced Under Slow Pyrolysis

**Biochar at 350°C**		**Groundnut shell**		**Shea nut shell**		**Groundnut and shea nut shells**	
**Mixture**	**Concentration**	***R*_i_**	**Int. effect**	***R*_i_**	**Int. effect**	***R*_i_**	**Int. effect**
***Cd*** *+ Hg*	0.08	80%	Ant	^*^	^*^	^*^	^*^
***Cd*** *+ Hg*	0.20	^*^	^*^	^*^	^*^	121%	Syn
***Hg*** *+ Cd*	0.20	80%	Ant	67%	Ant	9%	Ant
***Hg*** *+ Cd*	0.50	^*^	^*^	73%	Ant	214%	Syn
***Cd*** *+ Pb*	0.08	3050%	Syn	^*^	^*^	939%	Syn
***Cd*** *+ Pb*	0.20	^*^	^*^	^*^	^*^	117%	Syn
***Hg*** *+ Pb*	0.10	64%	Ant	^*^	^*^	^*^	^*^
***Hg +*** *Pb*	0.20	^*^	^*^	33%	Ant	^*^	^*^
***Hg*** *+ Pb*	0.50	60%	Ant	7%	Ant	86%	Ant
***Pb*** *+ Hg*	0.10	^*^	^*^	^*^	^*^	216%	Syn
***Cd*** *+ Hg + Pb*	0.08	2150%	Syn	^*^	^*^	121%	Syn
***Cd*** *+ Hg + Pb*	0.20	^*^	^*^	^*^	^*^	120%	Syn
***Hg*** *+ Cd + Pb*	0.10	^*^	^*^	^*^	^*^	191%	Syn
***Hg*** *+ Cd + Pb*	0.20	^*^	^*^	1333%	Syn	^*^	^*^
***Hg*** *+ Cd + Pb*	0.50	^*^	^*^	3333%	Syn	1143%	Syn
***Pb*** *+ Cd + Hg*	0.20	^*^	^*^	^*^	^*^	224%	Syn

**Note:**

^*^ Denotes adsorption was complete (100%) and R_i_ values cannot be computed for interpretation; all concentration values are in mg/l.

Abbreviations: Int. effect, Interactive effect; Ant, Antagonistic; Syn, Synergistic; R_i_, Relative adsorption capacity

**Table 8:** Interactive Effect of Mixture of Metal Ions in the Binary and Ternary Systems onto Biochars Produced Under Fast Pyrolysis

**Biochar at 700°C**		**Groundnut shell**		**Shea nut shell**		**Groundnut and shea nut shells**	
**Metal system**	**Concentration**	***R*_i_**	**Int. effect**	***R*_i_**	**Int. effect**	***R*_i_**	**Int. effect**
***Cd*** *+ Hg*	0.08	27%	Ant	^*^	^*^	^*^	^*^
***Hg*** *+ Cd*	0.10	350%	Syn	^*^	^*^	^*^	^*^
***Hg*** *+ Cd*	0.20	33%	Ant	^*^	^*^	^*^	^*^
***Hg + Cd***	0.50	27%	Ant	^*^	^*^	^*^	^*^
***Hg*** *+ Pb*	**0.20**	^*^	^*^	200%	Syn	^*^	^*^
***Hg*** *+ Pb*	**0.50**	^*^	^*^	62%	Ant	69%	Ant
***Cd*** *+ Hg + Pb*	**0.08**	35%	Ant	^*^	^*^	^*^	^*^
***Cd*** *+ Hg + Pb*	**0.20**	38%	Ant	^*^	^*^	^*^	^*^
***Hg*** *+ Cd + Pb*	**0.10**	1050%	Syn	^*^	^*^	^*^	^*^
***Hg****+ Cd + Pb*	**0.20**	67%	Ant	^*^	^*^	^*^	^*^
***Hg****+ Cd + Pb*	**0.50**	^*^	^*^	123%	Syn	154%	Syn

**Note:**

^*^ Denotes adsorption was complete (100%) and R_i_ values cannot be computed for interpretation; all concentration values are in mg/l.

Abbreviations: Int. effect, Interactive effect; Ant, Antagonistic; Syn, Synergistic

## Discussion

### Adsorption of mono-component system onto biochars

The experiment showed that shea nut shell biochars produced at 350 ± 5°C and 700 ± 5°C have the strongest affinity for Cd in the mono aqueous phase, followed by biochar that is a combination of groundnut and shea nut shells. Although the removal rate was generally effective, groundnut shell biochar produced at 700 ± 5°C and the combination of groundnut and shea nut shells produced at 350 ± 5°C were less effective in Cd removal at different high maximum contamination limits. This is probably due to the nature of feed stock. Shea nut shell biomass may be richer in lignocellulosic biomass (cellulose, hemicellulose and lignin), which is a good characteristic for effective adsorption of metal ions, hence the difference in their performance.

The present study found that groundnut and shea nut shell biochars produced at 350 ± 5°C and 700 ± 5°C have a strong affinity for Hg in the mono aqueous phase. Similarly, soybean stalk biochar was able to remove Hg by 75 to 87% from a mono aqueous solution.^23^ This experiment showed that high efficiency and cost-effective biochars can be derived from groundnut and shea nut shells as they require no pretreatment or modification of biochar surfaces for adsorption.

The results indicated that shea nut shell biochars produced at 350 ± 5°C and 700 ± 5°C had the strongest affinity for Pb in the mono aqueous phase, followed by groundnut shell biochar and the lowest involved the combination of groundnut and shea nut shell biochar. The individual groundnut and shea nut shell biochars had a high affinity for Pb in the mono-component system.

Similar agricultural wastes used for mono-component system studies on Pb showed efficiencies of 85% for chaff, 86% for sun flower husk, 90% for rice husk, 98% for tea waste and 100% for sesame husk for Pb ion removal.^24^ It has been reported that 32.80% to 42.30% adsorption of Pb onto biochar is often influenced by the coordination of the carbonyl and hydroxyl functional group.^25^ The experiment demonstrated that minerals in the groundnut and shea nut shells biochars may have contributed to the effective removal of trace metal ions from aqueous solutions.

### Adsorption of binary-component system onto biochars

A polluted aqueous environment may contain more than one metal ion. Therefore, remediation studies need to examine multiple metal interactions simultaneously for accurate adsorption of contaminants. Shea nut shell biochar produced at 350 ± 5°C and 700 ± 5°C have the strongest affinity for Cd and Hg ions in the aqueous phase, followed by biochar that involves the combination of groundnut and shea nut shell biochars.

The present study found that shea nut shell biochar produced at 350 ± 5°C and 700 ± 5°C had the strongest affinity for Cd and Pb ions in the binary aqueous phase. There was no clear difference between groundnut shell biochar and the combination of groundnut and shea nut shell biochar in terms of their removal efficiency. The results showed the order of adsorption to be Pb^2+^ > Cd^2+^ for Pb and Cd ions onto groundnut and shea nut shell biochars. This may be attributed to the fact that in the multi-metal adsorption isotherm, the hydrated radius of Pb^2+^ (4.01 A°) is smaller than that of Cd^2+^ (4.26 A°) and Pb has a greater affinity for most functional groups in organic matter, including phenolic and carboxylic groups.

The shea nut shell biochar produced at 350 ± 5°C and 700 ± 5°C had the strongest affinity for Pb and Hg ions in the aqueous phase, and the groundnut shell biochar produced at 350 ± 5°C had a slightly lower affinity for mercury compared to the combination of groundnut and shea nut shell biochars produced at 350 and 700°C, and groundnut shell biochar produced at 700°C.

### Adsorption of ternary-component system onto biochars

The experiment showed an order of adsorption for Cd, Hg and Pb ions onto groundnut and shea nut shell biochars in the ternary system of Pb^2+^> Hg^2+^ > Cd^2+^. An explanation for the trend in the ternary system is that the hydrated radius of Pb^2+^ (4.01 A°) is smaller than that of Hg^2+^ (4.22 A°) and Cd^2+^ (4.26 A°), and Pb has a greater affinity for most functional groups in organic matter. The ionic properties of the three metals make Pb more favorable in terms of adsorption through inner sphere surface complexation or sorption reactions compared to Hg and Cd. High adsorption of Pb^2+^ from aqueous solutions onto absorbents through surface electrostatic attraction is attributable to its high electronegativity constant of 2.33, which results in a high tendency for specific adsorption.^26^ The electrochemical potential, ionic charge and ionic radius affect adsorbent adsorption capacity in the multi-metal system.^27^ The adsorption capacity of biochars did not show any decrease or increase in any of the batch experiments due to the high affinity of biochars for trace metal ions that did not fully occupy the binding sites. The adsorption capacity of the multi-metal adsorption system decreases with respect to single metal adsorption capacity if the binding sites are fully occupied with remaining trace metals ions in the aqueous phase.^28^

### Adsorption performance of biochars produced at 350 ± 5°C and 700 ± 5°C

The present study found that shea nut shell-based biochar has a greater affinity for trace metals in the mono, binary and ternary systems. The combined biochars also seem to be better than biochar produced from groundnut shells. The study revealed biochars produced at 700 ± 5°C have greater affinity for metals in the aqueous phase, particularly in binary and ternary systems compared to biochars produced at 350 ± 5°C (slow pyrolysis). Fast pyrolysis temperatures and some types of biochar showed a slight increase in the adsorption efficiency of metal ions, but this increase was not statistically significant (p > 0.05). This implies that an increase in pyrolysis temperature did not significantly affect the mono, binary and ternary aqueous phases of Cd, Hg, and Pb ion adsorption onto biochar. This finding is supported by the findings of Kim et al. and Tran et al., who demonstrated the influence of pyrolytic temperatures of 300°C, 400°C, 500°C and 600°C, and 400°C, 500°C, 600°C, 700°C and 800°C on the adsorption of Cd from an aqueous solution onto giant miscanthus- and orange peel-derived biochar, respectively.^29^,^30^ It is clear that the properties of biochar influence their adsorption ability toward various pollutants and are affected by the nature and type of feed stock and pyrolysis conditions. Therefore, it is critical to manage appropriate conditions in the production of biochar.^9^

### Influence of Aqueous Phase pH on the Adsorption Capacity of Biochars

The influence of pH on adsorption largely depends on biochar type and the target metal ion. pH affects not only the biochar surface charge, but also the degree of ionization and speciation of the trace metal (adsorbate).^31^ The adsorption of trace metals was generally effective under the varied pH values of the aqueous phase. Adsorption was highly favored by the pH of the aqueous phase under the various maximum concentration limits, leading to the increase in Cd, Hg and Pb ion uptake. Generally, there is no significant difference (p > 0.05) in terms of difference in the pH of the aqueous phase in metal ion adsorption regardless of the component system and fold of the contamination limits. The present study suggests that the slight differences in the optimal pH for different metal ions in mono, binary and ternary systems may be due to the different solution chemistry of cadmium, mercury and lead ions.^32^

### Langmuir, Freundlich and Temkin isotherms

The Langmuir isotherm is valid for monolayer adsorption onto a surface containing a finite number of identical sites. The study results revealed that the Langmuir adsorption isotherm was the best model for trace metal ions adsorption onto biochars in batch experiments. The present study showed that Hg^2+^ had highest maximum uptake capacity onto SB350 and SB700. The present study found the order of maximum adsorption capacity to be mono > binary > ternary system. The 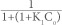 (R_L_), which is a dimensionless constant denoted as equilibrium parameter or separation factor, was found to be 0 < R_L_ < 1 regardless of the trace metal, biochar type or pyrolysis temperature, implying that nature of the adsorption was favorable. It can be concluded that the adsorption of the trace metal ions in this study is a chemical process.

The Freundlich isotherm was used to represent adsorption of Cd, Hg and Pb from the prepared aqueous phase onto biochars. It is a semi-empirical equation used to describe the multi-layer sorption and surface sorption under different non-ideal conditions.^33^ The two isotherm models were fitted in the adsorption of Cd, Hg and Pb by biochars produced from groundnut and shea nut shells. Generally, almost all values of 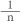 were less than 1, signifying adsorption by heterogeneous media where high energy sites were occupied first, before adsorption at lower energy sites.^34^ This is due to the low concentrations of toxic metals and indicates that the ions contain polar functional groups. It has been hypothesized that at low concentrations, ions in the aqueous phase are in competition for adsorption sites of biochar. However, 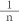 values for Hg and Cd were above 1 which indicates that a cooperative adsorption occurred.

The Temkin isotherm has a factor that explicitly takes into the consideration biochar (adsorbent) and metal ion (adsorbate) interactions. The Temkin adsorption isotherm used as the model assumes low interactions between the biochar (adsorbent) and trace metal ion (adsorbate) and energy of adsorption of all the molecules in the surface layer decrease at the cover surface.^35^ Generally, most of the co-efficient correlation values showed poor linearity regardless of the maximum capacity of adsorption used in the estimation of the coverage area. The few that were higher show good linearity regardless of the maximum capacity of adsorption used to for the estimation of the coverage area. It was clear that most of the correlation coefficients (R^2^) values were the poorest fit for the experimental data. Low values of B (constant related to heat of sorption (J/mol)) in this study suggest a weak interaction between biochars and metal ions supporting a mechanism of ion exchange. It also implies that the interaction between biochar and Cd, Hg and Pb ions in batch systems in the surface layer decrease at the cover surface.

### Interactive behavior of cadmium, mercury and lead ions in binary and ternary systems

The selectivity of biochars for Cd, Hg and Pb in binary and tertiary mixtures was determined in terms of relative metal *i* adsorption (R_i_ (%)) as defined by Chang and Chen.^22^ The R_i_ percentage was used to determine whether the interactions among mixtures in the binary and tertiary systems were antagonistic, synergistic or non-interactive in nature. The study revealed that interaction behaviors between the Cd, Hg and Pb ions were both antagonistic and synergistic in nature. For metal ions that are antagonistic in nature, their effect in the mixture is less than the sum of the individual effects of the constituents, or more likely, their individual effect on the substances added together is less than the expected response to multiple components. The antagonistic nature of some of the metal ions in the mixtures is attributable to the screening effect by the metals present in the solution.^36^ However, the synergistic nature of some of the metal ions in the present study, particularly mercury ions, suggests that their effect in the mixture is greater than the sum of the individual effect of the constituents or their individual effect on the substances added together is greater than the expected response to a multiple component system. Similarly, a multi-metal study involving metals such as nickel (II), Cd (II) and chromium (VI) by Jain et al. reported R_i_ values that were all less than 100% in binary and ternary metal systems.^37^ This suggests that the interactions between different metal ions are antagonistic in nature.

## Conclusions

The present study aimed to explore cost-effective biochars for adsorption of trace metals in an aqueous phase. The present study showed that shea nut shell-based biochar has a greater affinity for trace metals in mono, binary and ternary systems. The combined biochars performed better than biochar produced from groundnut shells only. The adsorption of trace metals was generally favored by various pH levels of the aqueous phase. The results revealed that Langmuir adsorption isotherm was the best model for the trace metal ions adsorption onto biochars in the batch experiment. The interaction behavior of the metals in the binary and ternary mixtures was either antagonistic or synergistic in nature. Fast pyrolysis temperatures (700 ± 5°C) and some types of biochar showed a slight increase in the adsorption efficiency of metal ions, but this increase was not statistically significant (p > 0.05). The results of the present study indicate that groundnut and shea nut shell-based biochars have a strong affinity for Cd, Hg and Pb ions in the aqueous environment. The development of biochar technology using groundnut and shea nut shells can satisfy the need for cost-effective and eco-friendly adsorbents for removal of aqueous trace metals. It is therefore recommended that further competitive adsorption studies of biochars be conducted for accurate estimation of adsorption in natural environments.
